# Genome-enabled prediction of quantitative traits in chickens using genomic annotation

**DOI:** 10.1186/1471-2164-15-109

**Published:** 2014-02-07

**Authors:** Gota Morota, Rostam Abdollahi-Arpanahi, Andreas Kranis, Daniel Gianola

**Affiliations:** 1 Department of Animal Sciences, University of Wisconsin-Madison, Wisconsin, USA; 2 Department of Animal Science, University College of Agriculture and Natural Resources, Karaj, Iran; 3 Aviagen, Midlothian, UK; 4 The Roslin Institute and Royal (Dick) School of Veterinary Studies, University of Edinburgh, Midlothian, UK; 5 Department of Biostatistics and Medical Informatics, University of Wisconsin-Madison, Wisconsin, USA; 6 Department of Dairy Science, University of Wisconsin-Madison, Wisconsin, USA

**Keywords:** Whole-genome prediction, Annotation, SNP, Chicken

## Abstract

**Background:**

Genome-wide association studies have been deemed successful for identifying statistically associated genetic variants of large effects on complex traits. Past studies have found enrichment of trait-associated SNPs in functionally annotated regions, while depletion was reported for intergenic regions (IGR). However, no systematic examination of connections between genomic regions and predictive ability of complex phenotypes has been carried out.

**Results:**

In this study, we partitioned SNPs based on their annotation to characterize genomic regions that deliver low and high predictive power for three broiler traits in chickens using a whole-genome approach. Additive genomic relationship kernels were constructed for each of the genic regions considered, and a kernel-based Bayesian ridge regression was employed as prediction machine. We found that the predictive performance for ultrasound area of breast meat from using genic regions marked by SNPs was consistently better than that from SNPs in IGR, while IGR tagged by SNPs were better than the genic regions for body weight and hen house egg production. We also noted that predictive ability delivered by the whole battery of markers was close to the best prediction achieved by one of the genomic regions.

**Conclusions:**

Whole-genome regression methods use all available quality filtered SNPs into a model, contrary to accommodating only validated SNPs from exonic or coding regions. Our results suggest that, while differences among genomic regions in terms of predictive ability were observed, the whole-genome approach remains as a promising tool if interest is on prediction of complex traits.

## Background

High-throughput genotyping technology has increasingly produced more dense sets of genetic markers, e.g., from tens to hundreds of thousands of SNP variables. Availability of high-density DNA genotyping chips, such as 770K and 600K SNP arrays in cattle [1] and chickens [2], respectively, are some recent examples. Also, sequencing of livestock species and humans (e.g., [3]) has revealed that coding DNA sequences (CDS) cover only a tiny fraction of the entire genome. A question of interest is that of estimating effects of non-coding sequences that are functional and could potentially influence phenotypes of interest.

Genome-wide association studies (GWAS) have been deemed successful for identifying statistically associated allelic substitution effects in known protein-coding genes. However, about 90% of trait-associated SNPs reported in humans do not lie within coding regions [4,5]. Hindorff et al. [4] found that nonsynonymous sites and 5Kb promoter regions were overrepresented in trait-associated SNPs, while depletion was observed for intergenic regions (IGR). Similar reports claiming enrichment within genic regions can be found in e.g., Knight et al. [6], Kindt et al. [7] and Schork et al. [8]. On the other hand, a recent release of the ENCyclopedia of DNA Elements (ENCODE) includes evidence of biochemical activity of the human genome [9]. About 62% of the genome is transcribed into RNA, and together with evidence such as transcription-factor-binding, specific chromatin structure and histone modification, the picture suggests that 80% of the human genome is involved in biochemical activity [9]. This implies that intergenic regions are likely to play important roles in complex traits. DNase hypersensitivity sites tagged by open chromatin indicate presence of an active regulatory role, and these are mostly located in intergenic and intronic regions (e.g., [10,11]). Further, a recent study suggests that more than 75% of identified SNPs are located in regulatory regions or are in strong linkage disequilibrium (LD) with SNPs in regulatory DNA segments [12]. There is also increasing evidence in animals that many markers found to be associated with traits of interest in GWAS reside in non-coding regions or gene deserts (e.g., [13,14]). Presumably these variants fall within *cis*-acting regulatory elements of genes residing nearby. Several authors have found a significant overlap between expression quantitative trait loci studies and genomic regions having effects in GWAS [6,7,15].

The preceding suggests that prediction of phenotypes from genomic information may not be as straightforward as commonly thought. For example, many SNPs not reaching stringent statistical significance criteria do contribute to additive genetic variance (e.g., [16]). Further, Eleftherohorinou et al. [17] reported a case where non-significant GWAS markers attained a better performance than that from use of significant GWAS markers alone when predicting rheumatoid arthritis. These studies clearly support the view that complex traits, often characterized as polygenic or as possessing an “infinitesimal” genetic architecture, are influenced by most genetic variations in the genome, with effects that may be too small to be detected with standard GWAS.

In light of the recent availability of SNP annotation information, it seems worthwhile to investigate genomic regions playing an important role in prediction of genetic values or phenotypes using high density SNP arrays. Whole genome-enabled prediction is currently applied to a wide range of agricultural species (e.g., [18]) and more recently to personalized medicine in humans [19]. Here, we used a whole-genome approach to prediction of phenotypes of commercial broiler chickens. The most common statistical model employed in this domain incorporates all available quality filtered SNPs into a linear regression model, contrary to accommodating only validated SNPs from exonic or coding regions. As an alternative, we examined partitioning SNPs based on their annotation, to characterize genomic regions that convey low or high predictive power. For instance, genic regions can be classified into CDS, 5’ and 3’ untranslated regions (UTR), exons, genes, introns, proximal regulatory regions, and non-genic regions, such as IGR.

The aim of this study was to evaluate and characterize the relative importance of genomic segments as contribution to predictive performance of phenotypes in chickens. The remainder of this paper is structured as follows. In the Methods section we present background on three chicken production traits and of the high dimensional SNP genotypes assessed on individual birds. This is followed by a description of SNP annotations and of the genome-enabled prediction model used in the study. Finally, results are presented and implications of the findings are discussed.

## Methods

Live animals were not used in this study and required no ethical approval. A sample from a commercial broiler chicken line consisting of 1,351 birds was provided by Aviagen. Three traits, body weight at 35 days (BW), ultrasound area of breast meat (BM) and hen house production (HHP, the total number of eggs laid between weeks 28 and 54) were available for 1,351, 1,336, and 823 animals, respectively. These animals were genotyped with the publicly available Affymetrix 600K chip, with information on 580,954 bi-allelic SNPs [2]. The chicken genome is comprised of 39 pairs of chromosomes: 5 pairs of macro-chromosomes, 5 pairs of intermediate size chromosomes, 28 pairs of micro-chromosomes, and sex chromosomes, Z and W [3]. The 600K SNP array includes SNPs mainly from chromosomes 1–28 and Z. Each SNP genotype was coded as 0 for homozygotes, 1 for heterozygotes and 2 for the alternative homozygotes. We applied the following editing criteria for data preprocessing: all SNPs with a call rate ≤ 95% and a minor allele frequency ≤ 1% were removed. Animals with fewer than 90% of SNPs genotyped were omitted. Missing genotypes were imputed independently locus by locus by sampling alleles twice from a Bernoulli distribution with probability equal to its observed allele frequency. This imputation strategy assumes Hardy-Weinberg equilibrium at a locus in question, as well as linkage equilibrium. Heritability estimates of these traits from this dataset were 0.30, 0.33, and 0.19 for BW, BM, and HHP respectively [20].

### SNP annotation

Chromosome information and physical positions of SNPs were obtained using the annotation file downloaded from the NetAffx website. We mapped the information to *Gallus_gallus_4.0* assembly through Ensembl database (release 71). Each SNP was examined to see if it resided in genic or non-genic regions. Five genomic regions were formed, namely, CDS, Exons (CDS + UTR), Genes (CDS + UTR + introns), Genes1kb (genes with regulatory regions), and intergenic SNPs which lie in remaining regions scattered all over the genome. CDS entail actual protein-coding sequences, whereas Exons further include UTR, Genes represent a combination of exons and introns, and Genes1kb incorporate nearby regulatory regions. Therefore, by definition, these genic regions present a nested structure. Regulatory regions were defined as 1kb upstream and downstream of genes, putatively *cis*-acting proximal genes. IGR in the present study consisted of SNPs without any assignment to the aforementioned annotation categories. Numbers of SNPs assigned to each of the genomic regions are shown in Table 1.

**Table 1:** Numbers of SNPs assigned to each genomic region

**Annotation**	**# of SNPs annotated**	**After filtering**
IGR	299,498	193,970
Genes ±1kb	281,455	184,047
Genes	266,947	183,768
Exons	29,764	19,511
CDS	21,975	14,416

IGR and CDS represent intergenic regions and coding DNA sequences, respectively.

### Whole-genome prediction models

We posited the phenotype of bird *i*, *y*_*i*_ (*i*=1,⋯,*n*) as a linear function of an intercept *μ*, a systematic effect *s*_*ij*_, a genetic effect *g*_*i*_, and a residual *ε*_*i*_, so that *y*_*i*_=*μ*+*s*_*ij*_+*g*_*i*_+*ε*_*i*_. Specifically, *s*_*ij*_ for BW and BM entailed a combined effect of sex, hatch week, contemporary group of parents and pen in the growing farm, whereas *s*_*ij*_ for HHP was a hatch effect. Here, *j* denotes the effect of level *j* of the corresponding group associated with bird *i*. If systematic effects are known to be present, one can fit these simultaneously with the genetic effect in the prediction model, or precorrect phenotype and use the residuals as a newly obtained phenotype.

The systematic effect on BW and BM had few replicates in each level. For instance, approximately 40% of the animals had a unique systematic effect, and 28% of birds had effects that were assigned only twice in the dataset. The number of levels for this factor was 908. This is common, e.g., in genetic evaluation of dairy cattle. A common strategy treats these effects as random by viewing levels as a random sample from a population (e.g., [21,22]). In this study, we preadjusted phenotypes for systematic effects by using a random effects model so that the model fitted was $y_{i}^{R}=\mu +g_{i}+e$ where $y_{i}^{R}$ represents precorrected phenotypes using the random model. The hatch factor for HHP contained 130 factor levels, and effects were also treated as random, as several levels were observed only once or twice.

To explore links between the aforementioned genomic regions and predictive power, the following comparisons were carried out. Predictive abilities of SNPs in each of the four genomic regions (CDS, Exons, Genes, Genes1kb) were compared with that from randomly sampled SNPs in IGR with an equal number of SNPs to those in the four regions. If a large number of regulatory elements is placed distantly from the genes that they regulate, or if influential regions span the entire genome but are not limited to particular segments, then IGR, devoid of protein-coding sequences, may have comparable or perhaps even better predictive power. On the other hand, if this does not hold, the collection of functionally enriched regions (e.g., CDS and Exons) would be expected to yield a better prediction than that delivered by SNPs in IGR. As a benchmark, a model using all available SNPs was tested as well.

### Bayesian ridge regression

Use of a semi-parametric kernel method for genome-enabled prediction was suggested first by Gianola et al. [23] and Gianola and van Kaam [24] in a mixed effects model context. Bayesian kernel ridge regression, a form of the Reproducing kernel Hilbert spaces methods, was entertained. Here, we present a succinct description of the kernel-based Bayesian ridge regression used. We postulated that the SNP-phenotype mapping for animal *i* is given by

(1) $$y_{i}^{R}=\mu +g(x_{i})+\varepsilon_{i},$$

where **x**_*i*_ is a vector of SNP genotypes observed on *i*. We assume **g** is represented as **K** ***α***, where **K** is an *n*×*n* kernel matrix indexed by the observed SNP covariates. This specification mitigates the “curse of dimensionality”, so that with **g**=**K** ***α***, the original 600K SNP predictors are reduced to the number of observations, that is 1,351, 1,336 or 823 animals. If we choose the residual sum of squares and the square of the norm of the coefficient ***α*** as a loss function and penalty, respectively, this is simply Bayesian ridge regression employing the kernel matrix **K** instead of the commonly used *n*×*p* genotypes matrix **X**, where *p* is the number of SNPs. We can now rewrite Equation (1) in matrix form, such that **y**= ***μ*** +**K** ***α*** + ***ε***. In order to implement the procedure under a Bayesian framework, a flat prior was assigned to ***μ***, and $\varepsilon ∼N(0,I\sigma_{\varepsilon }^{2})$, $\alpha ∼N(0,I\sigma_{\alpha }^{2})$ were assumed independent vectors. Scaled inverse chi-square distributions were assigned to the variance parameters $\sigma_{\varepsilon }^{2}$ and $\sigma_{\alpha }^{2}$, each with 3 degrees of freedom and a scale parameter equal to 1. Although this model makes use of kernels, it is different from the Bayesian kernel ridge regression applied by de los Campos et al. [25] and Morota et al. [26]. In our model, the penality takes the form *λ*|| ***α*** ||^2^, contrary to $\lambda ||K\alpha |{|}_{ℋ}^{2}$. Thus, the kernel matrix **K** is not included in the penalty function and optimization is not carried out under a Hilbert space. Our approach shares the spirit of that of Long et al. [27], where they regressed phenotypes on a kernel incidence matrix **K** by imposing an L1 regularization.

The kernel used was **K**∝**XX**^*T*^∝**G**, where **X** is a SNP genotype matrix as before, and **G** resulted from a centered and standardized **X**, followed by division by the number of SNP, as proposed by VanRaden [28]. This kernel is expected to capture genetic signals through genomic relationships under additive inheritance.

The Bayesian model was implemented by Gibbs sampling. For each genomic region, a MCMC chain was run and the first 20,000 samples were discarded as burn-in. Subsequently, 40,000 samples were obtained and thinned at a rate of 10, leaving 4,000 mildly correlated samples for posterior inference. Convergence of the chain was checked by visual inspection of trace plots of the parameters. The predictive ability of our Bayesian ridge regression model was assessed by a cross-validation (CV). Specifically, a 10 fold CV scheme was applied by assigning animals randomly to one of 10 disjoint subsets. Of these 10 subsets, 9 were combined to form a training set, and the remaining was used as testing set. Each of the 10 subsets was used as a testing set only once. Since the CV distribution was dispersed because of small sample size, the above 10 fold CV was replicated 15 times, at random. Predictive abilities were evaluated via the Pearson product-moment correlation between preadjusted phenotypes and predicted additive genetic values, that is $\text{cor}(y_{i}^{R},k_{i}^{T}{\hat{\alpha }}_{i})$, where $k_{i}^{T}$ is the *i*th row of **K**.

### Hierarchical clustering of predicted genetic values

Dissimilarities among various genomic regions were assessed using a hierarchical clustering method. For each trait, a matrix containing the pairwise Euclidean norms between predicted genetic values ($\hat{g}=K\hat{\alpha }$) obtained from different genomic annotations were calculated. This distance matrix was subsequently fed to the R function “hclust” for clustering purposes. Therefore, we classified genomic regions into hierarchical categories presumably sharing similar genomic signals captured by the kernel-based Bayesian ridge regression. At each iteration of the clustering algorithm, we joined the two most similar clusters, and distances between this newly merged cluster and each of the old clusters were computed by Ward’s criterion [29]. In Ward’s minimum-variance method, the distance between two clusters is defined as the increase in sum of squares between the two clusters provided that they are merged. The idea follows Heslot et al. [30], who investigated dissimilarities between various genome-enabled prediction models. However, our focus is on dissimilarities between genetic signals captured by several genomic regions.

## Results

**Figure 1:**
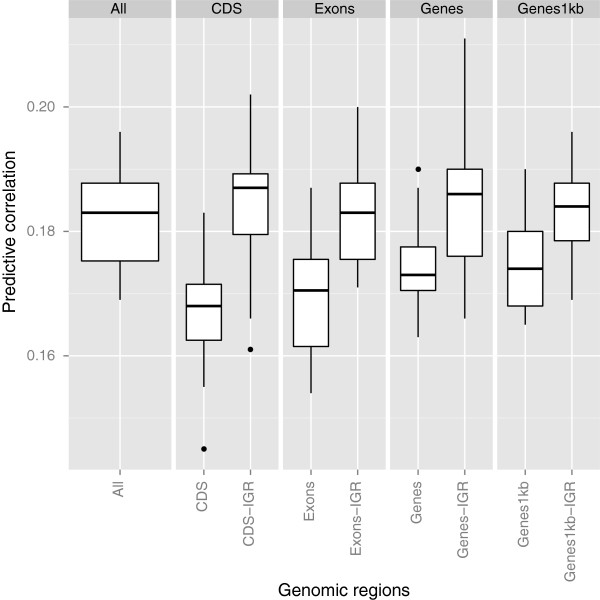
**Predictive correlations comparing genic and non-genic regions for BW using kernel-based Bayesian ridge regression.** The results were based on 10 fold cross-validation with 15 replications for each genomic region. Genic regions were coding DNA sequences (CDS), exons, genes, and genes with 1kb upstream and downstream. The genomic regions followed by the term “IGR” represent intergenic regions that contain equal SNP numbers to those of genic regions. “All” means all SNPs used for constructing **G**. Outliers denoted as black dots.

**Figure 2:**
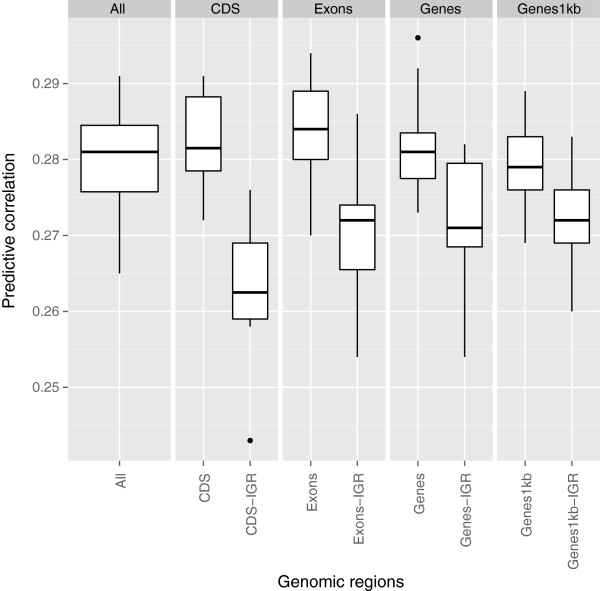
**Predictive correlations comparing genic and non-genic regions for BM using kernel-based Bayesian ridge regression.** The results were based on 10 fold cross-validation with 15 replications for each genomic region. Genic regions were coding DNA sequences (CDS), exons, genes, and genes with 1kb upstream and downstream. The genomic regions followed by the term “IGR” represent intergenic regions that contain equal SNP numbers to those of genic regions. “All” means all SNPs used for constructing **G**. Outliers denoted as black dots.

**Figure 3:**
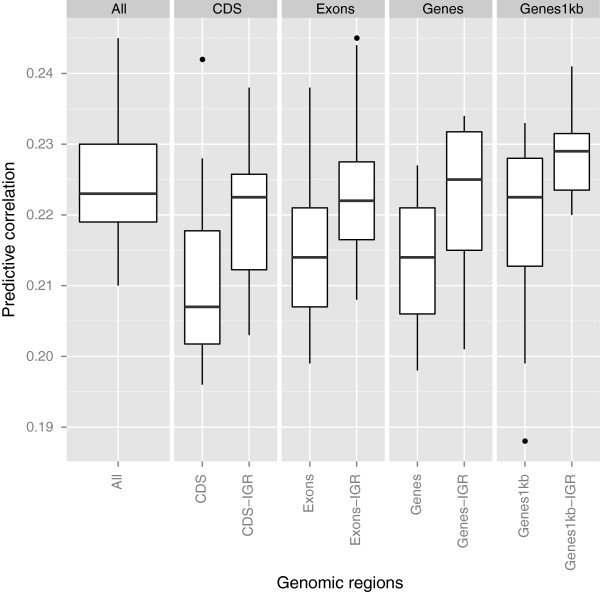
**Predictive correlations comparing genic and non-genic regions for HHP using kernel-based Bayesian ridge regression.** The results were based on 10 fold cross-validation with 15 replications for each genomic region. Genic regions were coding DNA sequences (CDS), exons, genes, and genes with 1kb upstream and downstream. The genomic regions followed by the term “IGR” represent intergenic regions that contain equal SNP numbers to those of genic regions. “All” means all SNPs used for constructing **G**. Outliers denoted as black dots.

Mean and median values of genomic relatedness (off-diagonals of **G**) between training and testing animals for each CV fold were negative or close to 0, regardless of a genomic region. Figures 1, 2, and 3 display predictive correlations obtained from the 10-fold CV with 15 replications, and these are summarized according to the annotation classes. Since CV variation across replicates was large because of small sample size, results were represented in boxplots. Figure 1 presents results for BW. Here, predictive power brought by SNPs in IGR was consistently better than for genic regions. Genetic signals were well tagged in IGR even when a small number of SNPs was considered simultaneously, as shown for the case of CDS. In IGR, performance with respect to prediction was similar irrespective of the number of SNPs assigned to classes. The additive genomic relationship kernel constructed from all markers attained a similar performance to that of IGR.

Results for BM presented a distinct pattern (Figure 2). Unlike BW, predictive abilities delivered by SNPs in genic regions were consistently better than for SNPs in IGR. We observed a slightly better predictive performance for CDS and Exons than for Genes and Genes1kb. The superiority of genic regions over IGR was most pronounced when their predictive abilities were compared to those of CDS-IGR and Exons-IGR. This suggests that SNPs in functionally enriched regions (e.g., exons) provide an important source of information for prediction of yet-to-be observed BM phenotypes. The predictive ability from SNPs in CDS was better than those for Genes1kb-IGR even though the two additive genomic relationship kernels constructed were from only 14,416 SNPs and 184,047 SNPs, respectively. In other words, close to 190,000 SNPs from IGR did not attain a similar predictive performance to that from CDS regions tagged by about 15,000 SNPs. Predictive ability delivered by all SNPs was similar to that of genic regions.

Figure 3 shows predictive correlations obtained for HHP. Results for HHP presented a similar pattern to BW, that is, IGR seemed able to convey power to the predictive model, with the corresponding SNPs likely to be scattered across the genome. This was evidenced by the lower correlations observed for genic regions. These results agreed with those for BW such that predictive performance of IGR was fairly constant, regardless of the number of SNPs considered. For both genic and IGR, the larger the number of markers, the greater the predictive correlations were. The picture that emerges is that SNPs in genic regions may carry genetic variations that are less useful for prediction of HHP than SNPs in IGR. Seemingly, the gain in prediction observed is not driven solely by functional genic regions but by IGR as well. Again, this may be partly attributed to the fact that IGR covers the entire genome.

**Figure 4:**
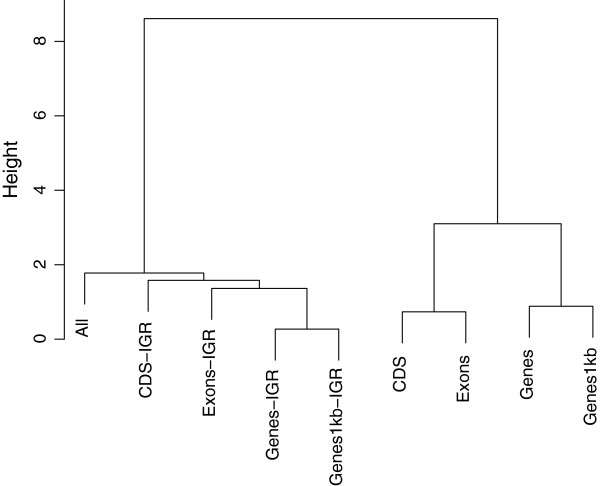
**Hierarchical clustering of predicted genetic values obtained from genic and non-genic regions for BW.** Genic regions were coding DNA sequences (CDS), exons, genes, and genes with 1kb upstream and downstream. The genomic regions followed by the term “IGR” represent intergenic regions that contain equal SNP numbers to those of genic regions.

**Figure 5:**
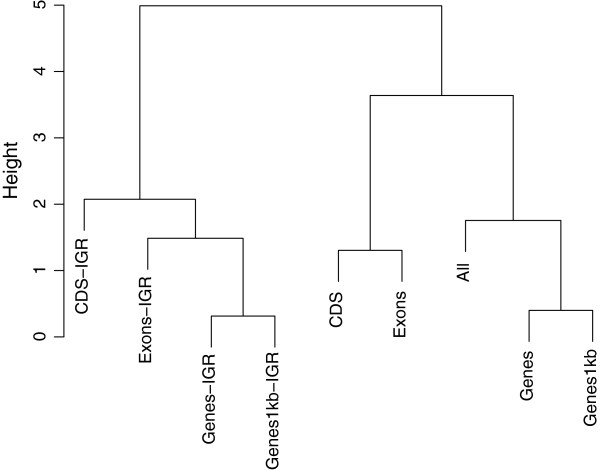
**Hierarchical clustering of predicted genetic values obtained from genic and non-genic regions for BM.** Genic regions were coding DNA sequences (CDS), exons, genes, and genes with 1kb upstream and downstream. The genomic regions followed by the term “IGR” represent intergenic regions that contain equal SNP numbers to those of genic regions.

**Figure 6:**
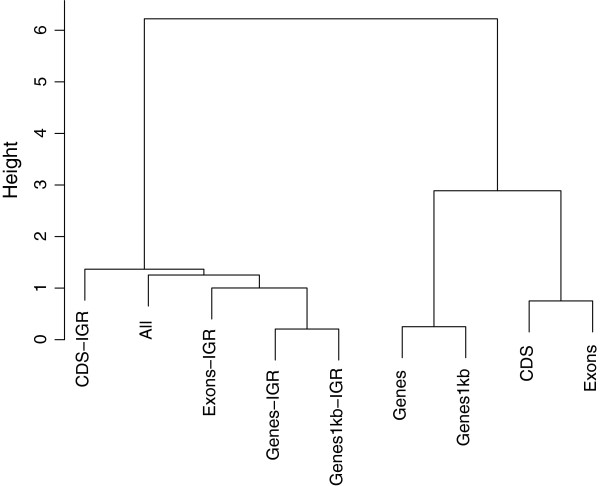
**Hierarchical clustering of predicted genetic values obtained from genic and non-genic regions for HHP.** Genic regions were coding DNA sequences (CDS), exons, genes, and genes with 1kb upstream and downstream. The genomic regions followed by the term “IGR” represent intergenic regions that contain equal SNP numbers to those of genic regions.

Results of the hierarchical clustering of predicted genetic values are in the dendrograms shown in Figures 4, 5, and 6. We took an agglomerative (bottom up) approach so that the most similar two clusters were combined into a higher-level cluster at each step until there was only one cluster left. In Figure 4, the top hierarchy on the dendrogram for BW was clustered by separating genic with non-genic regions. This is consistent with the boxplot of predictive correlations observed in Figure 1, genic and non-genic classes exhibited contrasting patterns. Genic and non-genic clusters were further subdivided based on CDS-Exons and Genes-Genes1kb. The dendrogram topology reflected the ability of genomic regions of capturing distinct types of genetic signals for prediction. While all available markers attained a predictive performance similar to those for all IGR, genetic values captured by all markers was clustered next to CDS-IGR.

For BM, the dendrogram had a slightly different structure as that of BW (Figure 5). Genic and IGR were grouped into different clusters, and this was in line with the results depicted in Figure 2, where genic regions consistently outperformed IGR. CDS-Exons and Genes-Genes1kb clustered with each other within subcategories. The genetic values obtained from all markers was clustered in the branch of genic regions, contrary to what was observed for BW.

Finally, the hierarchical clustering structure for HHP was similar to that of BW except in the case of CDS-IGR and Exons-IGR (Figure 6). It is interesting to note that Exon-IGR was clustered as more similar to Genes-IGR and Genes1kb than to CDS-IGR. For every comparison, predictive correlations from SNPs in IGR were larger than those from genic regions as displayed in Figure 3, and the dendrogram mirrored this pattern.

## Discussion

Advances in high-throughput genotyping technology have produced immense amounts of genetic data in livestock species and in humans. This has led to identification of significant trait-associated SNPs and to enrichment or depletion of these SNPs in annotated genomic regions. Seemingly, no systematic examination of links between genomic regions and predictive ability of complex phenotypes has been carried out up to date. We set out to associate SNP annotations with predictive performance using a commercial broiler chicken line genotyped with a 600K SNP array, to shed light on annotated interpretation of prediction performance. Attention was paid to identification of genomic regions that may deliver a high predictive ability for genome-enabled prediction of complex traits, with application in breeding and medicine.

There is debate on the role of functional regions in the genome in connection with what are called complex traits. Quantitative genetics theory claims that these traits are influenced by many genetic variations on the genome, with each of them having a small genetic effect [31]. Also, Wright [32] argued that pleiotropy is an universal phenomenon. On the other hand, presence of abundant biochemical activity at large proportion of the genome reported by the ENCODE project cannot be taken as solid evidence for claiming biological functionality [33].

We obtained annotation information from Ensembl to map SNPs to genic regions, and additionally considered all SNPs between 1kb upstream and 1kb downstream of genes. Predictive abilities delivered by genic regions and IGR varied between traits. It was found that some parts of the genome provided better predictive power than others. In particular, predictive performance for BM from genic regions marked by SNPs was consistently better than that of SNPs in IGR. For this trait, genic regions seemed to be enriched for variants that increase predictive ability, whereas the reverse was true for IGR. However, IGR tagged by SNPs were better than genic regions for BW and HHP. This highlights the importance of SNPs covering the entire genome, which implies that every allele may play a role in connecting phenotype with genotypes, albeit with a small contribution of individual loci. The usefulness of SNPs as genetic markers is that these span across the entire genome. This type of marker might be best suited to capturing genetic signals from widely distributed IGR. Presumably, structural variation data (e.g., copy number variations) in chicken will become available in the near future, but their contribution towards a better predictive performance may be limited for a trait like HHP, because such variations are observed only at particular regions in the genome. All genic regions considered delivered a better predictive performance for BM and an inferior prediction for BW and HHP.

Similarities between the genomic regions considered were investigated further using a hierarchical clustering method. Dendrogram topologies with genomic regions treated as clusters were consistent with results obtained in CV correlations. Seemingly, the hierarchical clustering agreed with the ability of genomic regions to deliver predictions for complex traits.

Previous studies have shown that many QTN (quantitative trait nucleotides) of large effects in animals tend to reside in coding regions, e.g., DGAT1 in cattle [34], but QTN in an intron of IGF2 in swine [35] and in IGR affecting stature in cattle [36] also exist. If a trait is controlled by SNPs that are not identified by GWAS or QTL analyses due to their small effect sizes, these SNPs are probably disseminated across the whole genome, potentially away from genic regions. On the other hand, if there are loci of large effect that exceed generally accepted genome-wide significance thresholds, these are likely to be found in genic regions. Therefore, it could be argued that, for prediction purposes, it may be crucial to consider IGR for complex traits, although this may be less important for regions with a major effect on phenotypes. If this is the case, BM may follow an oligogenic inheritance, while BW and HHP may conform to the assumptions of the infinitesimal model.

We conclude that examining sources of predictive performance aids in interpretation of results. Although genomic annotations of livestock species are more scarce than in humans, our approach may be adaptable to other traits and species as well. A recent study found that the GC content of CDS and introns was negatively correlated with gene expression levels in chicken, while 5’ UTR presented a positive association [37]. It is of interest to understand how the GC content of 5’ UTR could influence predictive performance of complex traits in future research.

Potential limitations of this study include that chromosome 16 was severely underrepresented due to scarce information on the current reference genome, and SNPs from chromosomes 29–38 were not available in the current SNP panels. Chromosome 16 contains the major histocompatibility complex, known to influence immune function [38]. The chicken has chromosomes differing markedly in length, and it is known that gene density of micro-chromosomes is much higher than in macro-chromosomes [3]. Also, note that the kernels constructed from five genomic regions may be also capturing signals from other regions because SNP genotypes are not orthogonal to each other, due to LD. Although presence of LD should not be ignored, our results indicate genetic signal tagging ability of SNPs in the genomic regions considered. In addition, IGR were simply defined as a collection of SNPs not residing in genes or 1kb upstream and downstream of genes. It may be interesting to further exclude known noncoding RNAs, transcription factor binding sites and microRNA binding sites in a future study. We also assumed, a priori, that genetic effects act independently and additively. However, there is growing evidence that a genetic signal is a product of a synergistic interplay of biological phenomena [39]. Hence, predictive models accommodating non-additive effects may provide additional insights. This work represents a first step toward examining sources of predictive performance of complex traits.

## Conclusion

Whole-genome prediction methods allow predicting complex traits, irrespective of knowledge of their molecular basis. Although this is typically regarded as a black box approach (e.g., [40]), dissection of available SNPs based on genomic annotation may be an attractive strategy for understanding which genomic segments drive higher predictive performance of yet-to-be observed phenotypes. We noted that predictive ability delivered by all markers was close to the best prediction achieved by the individual genomic regions. While a small difference among genomic regions in terms of predictive ability was observed, this suggests that whole-genome prediction methods are able to capture signals from the most useful genomic regions among several such sources. Thus, use of all markers seems the way to go, if interest is on prediction of complex traits.

## Competing interests

The authors declare that they have no competing interests.

## Authors’ contributions

GM conceived, carried out the study, and drafted the manuscript; RAA and AK provided critical insights and revised the manuscript; DG supervised the study and revised the manuscript. All authors read and approved the final manuscript.
